# Prognostic impacts of interstitial lung abnormalities on outcomes following resection for lung cancer

**DOI:** 10.1136/bmjresp-2024-002981

**Published:** 2025-08-28

**Authors:** Charles-Antoine Guay, Pierre-Emile Charest, Frederic-Thomas Caron, Louis Laflamme, Laurie Perreault, Anne-Sophie Laliberté, Elisabeth Albert, Genevieve Dion, Steeve Provencher

**Affiliations:** 1 Centre de Recherche de l’Institut universitaire de cardiologie et de pneumologie de Québec – Université Laval, Quebec City, Quebec, Canada; 2 Department of Medicine, Université Laval, Quebec City, Quebec, Canada; 3 Department of Radiology, Université Laval, Québec City, Quebec, Canada; 4 Department of Surgery, Université Laval, Québec City, Quebec, Canada; 5 Pulmonary Hypertension Research Group, Université Laval, Quebec city, Quebec, Canada

**Keywords:** Interstitial Fibrosis, Lung Cancer, Thoracic Surgery

## Abstract

**Introduction:**

The clinical significance of interstitial lung abnormalities in patients with lung cancer undergoing curative resection remains largely unstudied. This study aimed to evaluate the prevalence of these findings among patients with lung cancer undergoing resection and assess their impact on postoperative complications and long-term survival.

**Methods:**

This single-centre retrospective study included patients who underwent resection from 2008 to 2020. Patients with a history of lung cancer, previous lung resection or clinically evident interstitial lung disease before cancer detection were excluded. Preoperative chest scans were reviewed for interstitial lung abnormalities according to established criteria. Associations between these abnormalities and postoperative outcomes, as well as long-term survival, were analysed using multivariate models.

**Results:**

Among 1802 patients with available preoperative scans, 114 (6.3%) had interstitial lung abnormalities, including 17 (0.9%) with a usual interstitial pneumonia-like pattern. Interstitial lung abnormalities were associated with older age, female sex and smoking history. Although their presence did not significantly increase the risk of postoperative complications or 30-day mortality, interstitial lung abnormalities were linked to higher long-term mortality (92 vs 61 deaths/1000 person-years, HR 1.47; 95% CI 1.05 to 2.05). The usual interstitial pneumonia-like patterns were significantly associated with increased long-term mortality (HR 2.84; 95% CI 1.36 to 5.91), whereas other patterns were not (HR 0.98; 95% CI 0.63 to 1.54).

**Conclusions:**

Interstitial lung abnormalities are common in patients with lung cancer undergoing curative surgery and are linked to demographic and smoking-related factors. While they do not significantly impact short-term surgical outcomes, usual interstitial pneumonia-like pattern is associated with worse long-term survival.

**WHAT IS ALREADY KNOWN ON THIS TOPIC**

**WHAT THIS STUDY ADDS**

- This study evaluates the prevalence and prognostic significance of ILAs in patients with lung cancer undergoing curative resection, which was largely unstudied. It finds that ILAs are present in 6.4% of patients and do not significantly impact short-term postoperative outcomes, but ILAs with a fibrotic pattern are linked to higher long-term mortality.

**HOW THIS STUDY MIGHT AFFECT RESEARCH, PRACTICE OR POLICY**

- These findings highlight the need to explore management strategies to improve outcomes for patients with lung cancer and fibrotic ILAs.

## Introduction

 Interstitial lung abnormalities (ILAs) are a relatively new radiological entity representing incidental chest CT findings that may suggest interstitial lung disease (ILD) in patients without a clinical suspicion of ILD. ILAs have been defined radiologically as non-dependent abnormalities involving more than 5% of any lung zone,^1^ excluding centrilobular nodules and focal or unilateral patchy ground-glass opacities. A recent meta-analysis suggested a prevalence of 7% (95% CI 1% to 13%) among the general population, with a higher prevalence in older individuals, males and people who smoke.^2^ In addition, ILAs are increasingly being detected on chest CT scans performed for various purposes, such as lung cancer screening.^3 4^

The significance of ILAs remains to be determined, but there is increasing evidence that they are associated with adverse clinical outcomes. Indeed, recent cohort studies have documented that patients with ILAs are at increased risk of progressive ILD and lung malignancy, resulting in increased all-cause and cancer-related mortality compared with the general population.^5^,^7^ Importantly, ILAs are characterised by heterogeneous CT scan findings.^1^ However, increasing evidence suggests that subpleural fibrotic ILAs with a usual interstitial pneumonia (UIP)-like pattern are particularly associated with increased progression and worse survival.^3 8^

In many cases, ILAs are therefore expected to evolve into overt ILDs, including idiopathic pulmonary fibrosis (IPF). Due to overlapping risk factors,^1^ IPF is particularly common in individuals diagnosed with lung cancer.^5 9^ In addition, IPF is a significant risk factor for treatment-related complications and mortality following lung cancer resection.^10^ Conversely, only a limited number of studies have addressed whether ILAs influence postoperative complications and long-term survival in patients undergoing lung cancer resection.^6^ Therefore, we aimed to evaluate the prevalence of ILAs and their impact on postoperative complications and long-term survival in consecutive patients undergoing lung cancer resection.

## Methods

### Study population

Our research focused on patients with non-small cell lung carcinoma (NSCLC) who underwent lung resection at the Institut Universitaire de Cardiologie et de Pneumologie de Québec (IUCPQ) between 2008 and 2020. All patients undergoing their first surgery for lung cancer were considered eligible. Exclusion criteria included those with a history of lung cancer or previous lung resection, lack of available imaging data (see below) or those who received a diagnosis of lung metastasis, small cell lung cancer, carcinoid tumour or benign tumour during surgery. Patients known to have clinically evident ILD prior to the detection of lung cancer were also excluded. Of note, we estimate that over 95% of lung resections for cancer performed at our institution during this period were recorded in the biobank.

### Data collection

Data collection involved the extraction of relevant information from the biobank database. This included details such as sex, age, body mass index, smoking history (in pack-years), pulmonary function tests, extent and type of lung resection, histopathological diagnosis and tumour–node–metastasis staging based on the 2017 8th edition criteria with revisions for cases staged before 2017. Comorbidities, including chronic obstructive pulmonary disease (COPD, defined as a ratio of forced expiratory volume in 1 s (FEV1) to forced vital capacity (FVC) less than the lower limit of normal, calculated using the Global Lung Function Initiative equations^11^), coronary artery disease, peripheral artery disease, hypertension, dyslipidaemia, diabetes, connective tissue disease and chronic kidney disease, were prospectively determined by a careful review of medical records. Surgical details such as approach (eg, thoracoscopy vs thoracotomy), site of lung resection, length of hospital and intensive care unit stay, duration of pleural drainage and postoperative complications such as mechanical ventilation, pneumonia, pulmonary embolism, myocardial infarction, prolonged air leak (more than 7 days), respiratory failure (defined as hypoxemic or hypercapnic respiratory failure) and any other unspecified complication were meticulously documented from medical records and discharge summaries. Survival status through 31 December 2021 was obtained from the Régie de l'Assurance Maladie du Québec (RAMQ), a provincial organisation responsible for recording all deaths in the province of Québec.

### Chest CT analysis

Preoperative chest CT scans were reviewed by two chest radiologists with expertise in ILD who were blinded to clinical and pathological data, as well as previous imaging interpretations to determine which scans showed ILAs. A first radiologist (F-TC) identified patients with no evidence of ILA, intermediate ILA and overt ILA. CT scans with intermediate and overt ILA were then reviewed by an experienced thoracic radiologist (EA) to confirm the diagnosis. Discrepant results were resolved by consensus.

The diagnostic criteria for ILA were based on the definition proposed by the Fleischner Society.^1^ ILA was defined as incidental non-dependent changes involving more than 5% of any lung zone, including any combination of non-dependent ground-glass or reticular abnormalities, non-emphysematous cysts, honeycombing or traction bronchiectasis. CT scans with either focal or unilateral ground-glass attenuation, focal or unilateral reticulation, or patchy ground-glass abnormalities (<5% of the lung), which rarely represent an ILD, were not considered as significant ILA. Similarly, centrilobular nodules alone (typical presentation of smoking-related bronchiolitis) were excluded, as were patients whose lung abnormalities were likely due to previous treatments (resection, radiotherapy) or known complications (aspiration, recurrent pneumonia). The presence of reticular abnormalities, traction bronchiectasis, honeycombing and clustered cysts, contiguous walls and aeric attenuation (no normal lung parenchyma) was also detailed. ILAs were further characterised as a UIP-like ILA (encompassing both typical and probable patterns according to diagnostic criteria), defined as predominantly subpleural localisation and with evidence of architectural distortion with traction bronchiectasis or honeycombing (or both), whereas non-UIP-like ILA was defined as non-subpleural ILA or predominantly subpleural ILA without evidence of architectural distortion.^1^

### Outcomes

The primary outcome was overall survival after lung cancer resection, with the date of death serving as the endpoint. Secondary outcomes included postoperative complications within 30 days, further categorised into postoperative deaths, overall and respiratory-related complications. In addition, the assessment of conditional survival, defined as overall survival among patients who survived the first 30 days after lung cancer resection, was planned a priori to evaluate the association between ILAs and long-term outcomes excluding the impact of early postoperative mortality.

### Statistical analysis

Baseline characteristics before surgery were described, overall and by ILA status. Proportions were calculated for dichotomous variables, while means and SD were presented for continuous variables. Crude mortality rates were calculated for patients with ILA and for patients without ILA. Absolute risks (%) were obtained for 30-day postoperative complications. Survival analyses were performed to compare overall and conditional survival after a lung cancer resection in patients with UIP-like ILAs, non-UIP-like ILAs and patients without ILA. Unadjusted and adjusted HRs (aHRs) and corresponding CIs comparing mortality rates were estimated using Cox proportional hazards regression models. Risk ratios (RRs) for postoperative complications within 30 days by ILA type compared with patients without ILA were estimated using log binomial regression. Models were adjusted for age, sex, pack-years smoking, current smoking status, comorbidities, lung function, pathological type, surgical procedure and cancer stage. Model assumptions, including multicollinearity and the proportional hazards assumption, were examined and satisfied for each analysis. Complete data analyses were performed as missing data were minimal (<5%). We conducted stratified analyses according to COPD status and lung function metrics (FEV1, FVC and diffusing capacity of the lung for carbon monoxide (DLCO)) to explore the impact on postoperative and long-term outcomes. All analyses were performed using SAS statistical software (V.9.4; SAS Institute, Inc), with 95% CIs calculated using a 5% level of statistical significance. We did not adjust for multiplicity.

### Patient and public involvement

Patients and/or the public were not involved in the design, conduct or reporting of this research. The reporting of this study adhered to the Strengthening the Reporting of Observational Studies in Epidemiology guidelines.^12^

## Results

### Characteristics of the study population

Between 2008 and 2020, 2914 lung cancer resections were performed at the IUCPQ and recorded in the biobank. Of those, 1802 patients met our inclusion criteria. Baseline characteristics are shown in table 1. Excluded patients had fewer comorbidities and a lighter smoking history than included patients (online supplemental table 1). The ILA group comprised 114 (6.3%) patients, including 17 (0.9%) and 97 (5.4%) UIP and non-UIP-like ILAs, respectively. In univariate analysis, ILAs were associated with increasing age, increased proportion of females and increasing pack-years of smoking. Consistent with the slightly increased age, patients with ILAs also exhibited more comorbidities. They were also more likely to have decreased DLCO and squamous cell carcinoma.

**Table 1:** Characteristics of patients who underwent surgery for lung cancer between 1 January 2008 and 31 December 2020

	No ILA	ILA	Total	P value*
	N (%)	N (%)	N (%)	
Total	1688	114	1802	
Follow-up (years, mean±SD)	4.45±3.21	3.53±2.83	4.40±3.20	<0.0001
Age (mean±SD)	64.99±8.37	69.94±6.16	65.30±8.33	<0.0001
Sex				
Male	928 (54.98)	52 (45.61)	980 (54.38)	0.05
Female	760 (45.02)	62 (54.39)	822 (45.62)	
Ethnicity				
Caucasian	1549 (91.77)	108 (94.74)	1657 (91.95)	0.26
Other	139 (8.23)	6 (5.26)	145 (8.05)	
Comorbidity status				
Hypertension	855 (50.65)	66 (57.89)	921 (51.11)	0.13
Diabetes	254 (15.05)	20 (17.54)	274 (15.21)	0.47
COPD	992 (58.77)	71 (62.28)	1063 (58.99)	0.46
Coronary artery disease	312 (18.48)	37 (32.46)	349 (19.37)	0.0003
Chronic renal failure	65 (3.85)	5 (4.39)	70 (3.88)	0.77
Other comorbidities†	109 (6.46)	18 (15.79)	127 (7.05)	0.0002
Smoking status				
Never smoker	100 (5.92)	3 (2.63)	103 (5.72)	0.26
Former smoker	1235 (73.16)	92 (80.70)	1327 (73.64)	
Current smoker	353 (20.91)	19 (16.67)	372 (20.64)	
Pack-years of smoking				
Never smoker	100 (6.26)	3 (2.65)	103 (6.02)	0.02
1–20 pack-years	261 (16.24)	13 (11.50)	274 (16.02)	
21–40 pack-years	527 (33.00)	30 (26.55)	557 (32.57)	
>40 pack-years	709 (44.40)	67 (59.29)	776 (45.38)	
Pulmonary function test				
FVC≥80%	1319 (78.14)	91 (79.82)	1410 (78.25)	0.67
FVC<80%	369 (21.86)	23 (20.18)	392 (21.75)	
FEV_1_≥80%	955 (56.58)	72 (63.16)	1027 (56.99)	0.17
FEV_1_<80%	733 (43.42)	42 (36.84)	775 (43.01)	
DLCO≥80%	979 (58.00)	43 (37.72)	1022 (56.71)	<0.0001
DLCO<80%	709 (42.00)	71 (62.28)	780 (43.29)	
Pathological type				
Adenocarcinoma	1142 (67.65)	55 (48.25)	1197 (66.43)	<0.0001
Squamous cell carcinoma	314 (18.60)	43 (37.72)	357 (19.81)	
Other‡	232 (13.74)	16 (14.04)	248 (13.76)	
Cancer stage				
1	1048 (75.23)	70 (72.92)	1118 (75.08)	0.09
2	214 (15.36)	22 (22.92)	236 (15.85)	
3A	108 (7.75)	4 (4.17)	112 (7.52)	
3B/4	23 (1.65)	0 (0.00)	23 (1.54)	
Surgery type				
Pneumonectomy	116 (6.87)	6 (5.26)	122 (6.77)	0.76
Lobectomy/bilobectomy	1261 (74.70)	88 (77.19)	1349 (74.86)	
Sublobar resection	311 (18.42)	20 (17.54)	331 (18.37)	

* P values calculated using Pearson’s χ2 test, Fisher's exact test or the Wilcoxon rank-sum test.

† Other comorbidities include pulmonary embolism, pulmonary hypertension and connectivitis.

‡ Other pathological types include adenosquamous carcinoma, large cell carcinoma, basaloid carcinoma, sarcomatoid carcinoma, as well as mixed tumours (ie, multiple histological subtypes in the same lesion).

COPD, chronic obstructive pulmonary disease; DLCO, diffusing capacity of the lung for carbon monoxide; FEV_1_, forced expiratory volume in 1 s; FVC, forced vital capacity; ILA, interstitial lung abnormalities.

### Postoperative outcomes in relation to ILA

The length of hospital stay was similar in ILA and non-ILA patients (6.3±5.2 vs 6.5±6.4 days, p=0.64). Overall, a total of 21 (1.2%) patients died within 30 days of surgery, with the higher rates observed after pneumonectomy compared with other surgical approaches (data not shown). In addition, 736 (40.8%) patients had a postoperative complication within 30 days of the procedure, including 392 (21.8%) respiratory complications. The proportion of patients who died within 30 days of surgery was numerically higher in the ILA group (n=4/114 (3.5%) vs 17/1688 (1.0%)). However, this increase in postoperative death (RR 1.45; 95% CI 0.30 to 6.99, p=0.64) was not significant when adjusted for age, sex, pack-years of smoking, current smoking status, comorbidities, surgical procedure and cancer stage; and the presence of ILA was not associated with an increase in overall complications (RR 0.78; 95% CI 0.59 to 1.03, p=0.08) (table 2). Surprisingly, the presence of ILA was associated with a reduction in respiratory complications (RR 0.55; 95% CI 0.33 to 0.93, p=0.02).

**Table 2:** Relationship between 30 days postoperative complications of lung cancer surgery and imaging pattern: results from multivariate binomial regressions

	Death			All complications			Respiratory complications		
	n/N	aRR* (95% CI)	P value	n/N	aRR* (95% CI)	P value	n/N	aRR* (95% CI)	P value
Imaging pattern									
No ILA	17/1688	REF		692/1688	REF		374/1688	REF	
ILA	4/114	1.45 (0.30 to 6.99)	0.64	44/114	0.78 (0.59 to 1.03)	0.08	18/114	0.55 (0.33 to 0.92)	0.02
Non-UIP ILA	3/97	1.76 (0.37 to 8.28)	0.48	37/97	0.76 (0.56 to 1.03)	0.08	13/97	0.44 (0.24 to 0.82)	0.01
UIP ILA	1/17	NC	–	7/17	0.99 (0.55 to 1.77)	0.97	5/17	1.37 (0.60 to 3.14)	0.45

* Adjusted analyses adjusted for age, sex, pack-years smoking, current smoking status, comorbidities, lung function, surgery type and approach (thoracoscopy or thoracotomy), pathological type and cancer stage.

aRR, adjusted relative risk; ILA, interstitial lung abnormalities; NC, not calculable; REF, reference; UIP, usual interstitial pneumonia.

### Long-term outcomes in relation to ILA

**Figure 1:**
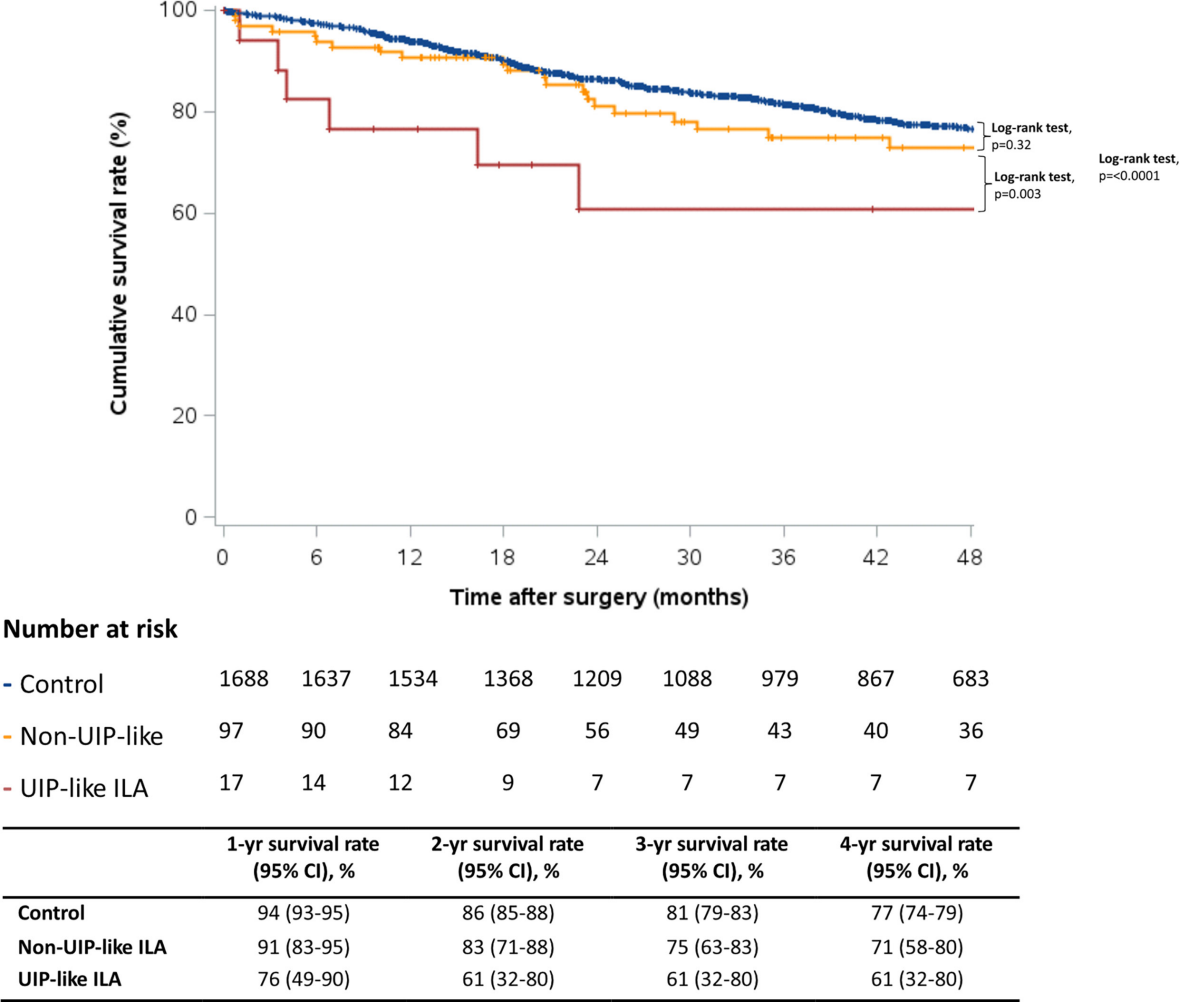
Kaplan-Meier survival plots of study subjects based on three comparison groups: controls, patients with non-usual interstitial pneumonia (UIP), interstitial lung abnormalities (ILAs) and patients with UIP-like ILA. The log-rank test was used to assess the differences between the survival curves.

There were 496 (27.5%) deaths during a mean follow-up period of 4.4±3.2 years. The presence of ILA was associated with an overall increase in long-term mortality compared with patients without ILA (92 vs 61 deaths/1000 person-years, HR 1.47; 95% CI 1.05 to 2.05, p=0.03 (table 3). However, using Cox proportional hazards models adjusting for age, sex, pack-years smoking, current smoking status, comorbidities, surgical procedure and cancer stage, only a UIP-like pattern remained significantly associated with mortality (207 deaths/1000 person-years, HR 2.84; 95% CI 1.36 to 5.91, p=0.005), whereas no significant increase in the risk of death was observed for non-UIP ILA (76 deaths/1000 person-years, HR 0.98; 95% CI 0.63 to 1.54, p=0.94). A Kaplan-Meier analysis of long-term survival in patients with UIP-ILA, non-UIP ILA and controls is shown in figure 1. To account for a possible impact of ILA on early postoperative mortality, sensitivity analyses were performed using conditional survival. When excluding patients who died within the 30-day postoperative period, UIP-like ILA remained associated with long-term mortality (online supplemental table 2).

**Table 3:** Association between imaging pattern and mortality in patients who underwent surgery for lung cancer: results from univariate and multivariate Cox proportional hazards models

	Deaths/person-years	Crude HR			aHR*		
		HR	95% CI	P value	aHR*	95% CI	P value
No ILA	459/7519.48	REF			REF		
ILA	37/402.36	1.47	1.05 to 2.05	0.03	1.19	0.80 to 1.76	0.39
Non-UIP ILA	27/354.16	1.22	0.83 to 1.80	0.32	0.98	0.63 to 1.54	0.94
UIP ILA	10/48.20	3.28	1.75 to 6.14	0.0002	2.84	1.36 to 5.91	0.005

* Adjusted analyses are adjusted for age, sex, pack-years smoking, current smoking status, comorbidities, lung function, surgery type and approach (thoracoscopy or thoracotomy), pathological type and cancer stage.

aHR, adjusted HR; ILA, interstitial lung abnormalities; REF, reference; UIP, usual interstitial pneumonia.

### Exploration of subgroups of interest

We then explored whether the association between ILAs and outcomes was predominantly observed in prespecified subgroups based on baseline pulmonary function tests. Interestingly, the association between ILAs and poorer long-term outcomes was predominantly observed among patients with UIP-like ILAs exhibiting poorer lung function, whereas concomitant lung obstruction did not influence this association (table 4 and online supplemental table 3).

**Table 4:** Association between ILA and long-term mortality in patients who underwent surgery for lung cancer according to (A) FVC and (B) DLCO

A	FVC≥80%		FVC<80%		aHR* (95% CI)
	Deaths/person-years	aHR* (95% CI)	Deaths/person-years	aHR* (95% CI)	for FVC within strata of ILA group
No ILA	335/6079.83	1	124/1439.65	1.15 (0.86 to 1.53)	1.15 (0.86 to 1.53)
ILA	33/321.25	1.67 (1.10 to 2.52)	4/81.11	0.28 (0.07 to 1.12)	0.17 (0.04 to 0.71)
Non-UIP ILA	24/273.35	1.50 (0.94 to 2.40)	4380.81	0.14 (0.02 to 0.99)	0.09 (0.01 to 0.69)
UIP ILA	9/47.90	2.55 (1.17 to 5.57)	1/0.29	65.96 (8.03 to 524.17)	25.86 (2.87 to 232.67)
**B**	**DLCO≥80%**		**DLCO<80%**		aHR* **(95% CI)**
	**Deaths/person-years**	aHR* **(95% CI)**	**Deaths/person-years**	aHR* **(95% CI)**	**for DLCO within strata of ILA group**
No ILA	207/4660.22	1	252/2859.26	1.39 (1.09 to 1.78)	1.39 (1.09 to 1.78)
ILA	9/171.56	1.01 (0.49 to 2.08)	28/230.80	1.69 (1.04 to 2.75)	1.68 (0.73 to 3.83)
Non-UIP ILA	9/165.03	1.05 (0.51 to 2.16)	18/189.13	1.27 (0.70 to 2.29)	1.21 (0.49 to 2.95)
UIP ILA	0/6.53	NC	10/41.67	3.74 (1.77 to 7.90)	NC

* Adjusted analyses are adjusted for age, sex, pack-years smoking, current smoking status, comorbidities, lung function, surgery type and approach (thoracoscopy or thoracotomy), pathological type and cancer stage.

aHR, adjusted HR; DLCO, diffusing capacity of the lung for carbon monoxide; FVC, forced vital capacity; ILA, interstitial lung abnormalities; NC, not calculable; UIP, usual interstitial pneumonia.

## Discussion

In this retrospective cohort study, we found pre-existing ILAs to be associated with reduced long-term survival in patients with lung cancer who underwent curative surgery between 2008 and 2020. Interestingly, the impact of pre-existing ILAs on postoperative long-term survival was largely heterogeneous according to imaging patterns. Exploratory analyses suggested this association was predominantly observed among patients with impaired preoperative lung function. Conversely, ILAs were not associated with significantly increased early mortality and complications, suggesting that the association between ILAs and long-term mortality is independent of the initial lung cancer management.

The present findings contribute to the growing body of evidence highlighting a connection between pre-existing ILAs and mortality linked to cancer.^1 2 7 13^ This correlation spans across patients at different stages of cancer treatment, from those undergoing surgical resection^2 5 6 14^ to those receiving systemic therapy.^15^ The exact cause behind this heightened mortality remains unclear; however, other research suggested that the risk of lung injury associated with ILAs and cancer treatments may play a significant role.^1 6^ Systemic therapies involving chemotherapy, targeted tyrosine kinase inhibitors, immunotherapy checkpoint inhibitors and antibody–drug conjugates are associated with an increased incidence of pneumonitis in patients with pre-existing ILAs.^16^,^18^ The presence of pre-existing ILAs also escalates the likelihood of severe radiation pneumonitis in patients with lung cancer undergoing stereotactic body radiotherapy.^19 20^ Additionally, surgical interventions for lung cancer have been linked to an increased risk of acute lung injury during the postoperative period.^1 6 14^

While plausible mechanisms that could explain worse outcomes in patients with pre-existing ILAs are rapidly emerging, evidence from real-world observational data is still needed to better understand the impact of pre-existing ILAs on survival beyond the immediate postoperative period.^21^ A recent matched case–control study found a significant increase in mortality rates in patients with ILAs who underwent surgical treatment for lung cancer in South Korea, but the authors were unable to assess the independent effect of specific ILA patterns or perform stratified analyses based on pulmonary function to better understand the impact of ILAs in specific subgroups of the population at higher risk of complications.^6^ Significant insights emerged from our study concerning the long-term implications of specific ILA patterns in patients undergoing curative surgery for NSCLC. Specifically, our subgroup analysis revealed that patients with a UIP-like pattern of ILAs had a significantly higher risk of long-term mortality (HR 2.84; 95% CI 1.36 to 5.91, p=0.005) compared with those without ILAs. This contrasts with non-UIP ILAs, which were not independently associated with unfavourable long-term survival outcomes. Similar results were obtained by Fujiwara *et al*, who found higher mortality rates in patients with stage I–III lung cancers with ILAs with a definitive or probable UIP pattern following lung resection.^22^ However, the authors were unable to adjust for important potential confounders, such as comorbidities, which share common risk factors with ILAs. This limitation may have resulted in either an overestimation or underestimation of the association between ILAs and mortality.^7^ Our findings are also consistent with the results of Axelsson *et al*,^5^ who reported higher mortality rates from lung cancer in patients with fibrotic ILAs compared with patients without definite fibrosis imaging pattern. This observation highlights the significant heterogeneity in the clinical significance of ILAs and encourages further exploration into potential explanations for the observed disparities in outcomes between UIP-like ILAs and non-UIP ILAs. One hypothesis is that the radiological similarities between UIP-like ILAs and IPF may imply a shared pathophysiological process.^8^ Supporting this hypothesis, it has been found that individuals with ILAs share a common syndrome observed in patients with IPF, which includes the development of a restrictive lung deficit, accelerated decline in lung function, imaging progression and reduced subjective health and physical function.^21^ Moreover, it has been shown that UIP-like ILAs share genetic determinants more closely linked to IPF compared with other ILA subtypes and patterns.^23^ Given that IPF is known for its progressive and fatal nature, the presence of a UIP-like pattern in ILAs could indicate a more advanced or aggressive form of interstitial lung pathology, predisposing patients to worse clinical outcomes. Further exploration into these hypotheses and the molecular mechanisms underlying UIP-like ILAs is warranted to elucidate their implications for patient care and prognosis. Interestingly, we found a relatively high proportion of patients in our cohort with a UIP-like subtype of ILA compared with the prevalence of IPF in the general population (0.007–0.042%).^24^ The higher prevalence of ILAs compared with IPF could provide the scientific community with a larger data set, enabling a better understanding of this rare but lethal disease. If ILAs represent an early stage in the progression towards IPF, this could suggest a promising potential window for introducing therapies aimed at slowing disease progression.

The shape of the survival curve is also of great interest for prognosis determination and treatment selection in patients with lung cancer. Indeed, reduced survival may be due to early postoperative mortality caused by increased complications, which could argue in favour of a less invasive treatment approach in more fragile patients such as patients with UIP-like ILAs. Interestingly, our results did not show statistically significant differences in short-term postoperative mortality or postoperative complications, which contrasts with previous studies. For example, a cohort study of 488 healthy elderly patients undergoing lung cancer surgery^14^ revealed that ILAs were associated with a higher risk of postoperative pulmonary complications (adjusted OR 1.91; 95% CI 1.14 to 3.21), and a matched case–control study comparing healthy patients to subjects with ILAs and IPF^6^ reported similar findings (OR 9.56; 95% CI 2.85 to 32.1). As in the present study, however, the CIs were wide, which prevented definitive conclusions about the extent of the increased risk of postoperative complications. Additionally, unlike the present study, the authors could not adjust for important confounders such as comorbidity status, lung function and cancer stage.^25 26^ These discrepancies might also arise from differences in cohort size and patient characteristics. We also found ILAs to be associated with reduced respiratory complications, which is in contrast with previous findings.^6 14 22^ This surprising result may indicate residual confounding inherent to observational studies, which may be exacerbated by the small number of respiratory complications in the ILA group. For example, a higher proportion of patients without ILAs had reduced preoperative lung function, as measured by FEV₁ and FVC, both of which are well-established predictors of postoperative respiratory complications.^27^,^29^ However, due to the limited number of complications observed in the ILA group, lung function had to be included as a dichotomous variable in the analysis. This constraint may have resulted in residual confounding, potentially contributing to the unexpected association observed.^30^ Nonetheless, while the overall association appears to be primarily driven by patients with non-UIP ILAs, an elevated, rather than reduced, risk of respiratory complications was also observed among patients with UIP ILAs in coherence with previous findings,^22^ although this association did not reach statistical significance. Despite similar complication rates, long-term survival of patients with a UIP-like ILA remains poor, which suggests that UIP-like ILAs could have a lasting effect on long-term survival following curative surgery for lung cancer. This is supported by sensitivity analyses of conditional survival. Furthermore, although exploratory, subgroup analyses also suggest that patients with mildly impaired lung function might be at particularly increased mortality rates. Interestingly, Fujiwara *et al* also reported that reduced preoperative DLCO was associated with a higher risk of severe acute postoperative complications in patients with lung cancer with ILAs compared with those with ILAs but preserved lung function. Although obtained from univariate analyses, this finding supports the validity of our subgroup analyses.^22^ In the absence of clinical evaluation, it is impossible to clearly distinguish between ILA and potentially clinically significant ILD in patients with impaired lung function, according to the classification proposed by the Fleischner Society.^1^ Nonetheless, if these findings are confirmed in other populations, they may help identify vital prognostic factors to consider before undertaking curative surgery in patients with lung cancer.

This work also revealed that a considerable proportion of patients with lung cancer who underwent curative surgery had pre-existing ILAs, with 114 out of 1802 patients (6.3%) meeting the definition proposed by the Fleischner Society.^1^ We observed associations between ILAs and several demographic factors, including advanced age and a history of smoking—factors known to contribute to both ILAs and lung cancer. In our cohort, we also observed a slightly higher proportion of females with ILAs. This finding contrasts with previously published cohorts, where the association between sex and ILA prevalence has been inconsistent. Some studies reported a higher prevalence among males,^6 22 31^ while others found it to be more common in females.^7 8^ This apparent discrepancy may be attributable to random variation, given the relatively small number of patients with ILAs across available cohorts. Alternatively, it may reflect sex-related differences in risk factors for ILA development, such as smoking habits and exposure to air pollution, which can vary across regions of the world.^32^

Interestingly, patients with ILAs did not experience a higher rate of immediate postoperative complications, suggesting that surgical treatment remains a viable option in this population. Conversely, prior research indicates that ILAs may increase susceptibility to adverse events from chemotherapy, immunotherapy and radiation.^16 19 20^ Quantifying the effect of ILAs on treatment outcomes could refine risk–benefit analysis and inform treatment choices. While our findings indicate persistent excess mortality in patients with UIP-like ILAs, the lack of cause-of-death data prevents definitive conclusions. Given the potential impact of ILAs on lung cancer treatment, further exploration is needed to understand how the presence of ILAs—especially those with a UIP-like pattern—may guide shared decision-making, especially in complex surgical cases or stage 3 NSCLC, which involves significant treatment heterogeneity.^33^

While the present study benefits from a substantial cohort size and a high participation rate, enhancing the reliability of our findings and allowing for more robust statistical analyses, notable limitations should be acknowledged. First, the retrospective design of this study restricts our ability to establish causality and may introduce biases related to data collection and patient selection. Second, although our larger sample compared with previous studies^6^,^1113 14^ allowed for in-depth stratified analyses, the still relatively small number of patients with ILAs may limit the generalisability of our findings and the statistical power to detect significant differences in certain outcomes. For example, our results might not be generalisable to patients of different ethnicities and with more advanced stages of cancer. Moreover, in the absence of more detailed data on genetic markers such as epidermal growth factor receptor (EGFR), anaplastic lymphoma kinase (ALK), and ROS proto-oncogene 1 (ROS1), we cannot exclude the possibility of heterogeneity based on driver mutations. Future studies should aim to explore this potential variability. Similarly, the limited number of postoperative complications observed in our cohort might have influenced our ability to identify meaningful associations between ILAs and short-term postoperative outcomes. Third, reliance on existing medical records and imaging data may lead to variability in the classification and identification of ILAs, potentially impacting the accuracy of our findings. We limited the possibility of misclassification bias by relying on double assessment by two chest radiologists blinded to the survival outcome and other clinical data. Nevertheless, CT imaging may have limitations in fully characterising early or subtle ILA, and histopathological correlation, although not feasible in the preoperative evaluation setting, could offer deeper insights into the underlying nature of ILAs. The incorporation of pathological specimens in future studies may help refine ILA subclassification and clarify the relationship between radiological patterns and histological diagnoses. Finally, in the absence of the causes of death in the RAMQ registry, we were unable to assess the impact of ILAs on specific causes of death, such as cancer progression or mortality related to respiratory conditions, which would have allowed for a better understanding of the observed trends. Despite these limitations, our study’s strengths lie in its comprehensive follow-up data, providing valuable insights into the long-term implications of ILAs in patients with lung cancer, as well as its rigorous methodology, including detailed radiographic assessments and adjustments for a large number of potential confounding variables limiting the potential for residual confounding, which strengthens the validity of our conclusions.

In conclusion, our investigation underscores the intricate relationship between ILAs and outcomes in patients undergoing curative surgery for lung cancer. While ILAs share similarities with IPF and independently contribute to increased lung cancer mortality risk, their clinical impact appears to differ based on radiological subtypes. Notably, UIP-like ILAs are associated with significantly higher long-term mortality, suggesting a need for subtype-specific management strategies. Future research should aim to elucidate the underlying mechanisms driving these associations in order to facilitate the development of targeted therapeutic approaches and refined prognostic models. Additionally, prospective studies with larger and more diverse cohorts are necessary to validate and expand upon our findings, ensuring that the nuances of ILA subtypes are accurately captured and addressed in clinical practice.

## Supplementary material

10.1136/bmjresp-2024-002981 online supplemental file 1

## Data Availability

All data relevant to the study are included in the article or uploaded as supplementary information. Additional data supporting the findings of this study, not shown in the article, are available from the corresponding author upon reasonable request.
