# Supplementary material for: Prognostic impacts of interstitial lung abnormalities on outcomes following resection for lung cancer
Source: BMJ Open Respir Res. 2025 Aug 28;12(1):e002981. doi: 10.1136/bmjresp-2024-002981 (PMC12410612; doi:10.1136/bmjresp-2024-002981)
Supplement: online supplemental file 1 [file bmjresp-12-1-s001.docx]

**PROGNOSTIC IMPACTS OF INTERSTITIAL LUNG ABNORMALITIES ON OUTCOMES FOLLOWING RESECTION FOR LUNG CANCER**

Charles-Antoine Guay, MD, MSc^1,2^*, Pierre-Émile Charest, MD^1^*, Frédéric-Thomas Caron MD^1^, Louis Laflamme, MD^1,2^, Laurie Perreault^1,3^, Anne-Sophie Laliberté, MD^1,4^, Elisabeth Albert MD^1,3^, Geneviève Dion, MD^1,2^, Steeve Provencher, MD, MSc^1,2,5^.

^1^ Centre de Recherche de l’Institut Universitaire de Cardiologie et de Pneumologie de Québec, Québec City, Québec, Canada.

^2^ Department of Medicine, Université Laval, Québec City, Québec, Canada.

^3^ Department of Radiology, Université Laval, Québec City, Québec, Canada.

^4^ Department of Surgery, Université Laval, Québec City, Québec, Canada.

^5^ Pulmonary Hypertension Research Group ([www.phrg.ca](http://www.phrg.ca))

*Co-first authors (equally contributed)

**Online Data Supplement**

**Table S1**. Characteristics of excluded patients who underwent surgery for lung cancer between January 1, 2008, and December 31, 2020.

|  | **Excluded** | **Included** | **P value^*^** |
| --- | --- | --- | --- |
|  | N (%) | N (%) |  |
| Total | 1,112 | 1,802 |  |
| Age (mean ± SD) | 64.98 ±8.50 | 65.30 ±8.33 | 0.31 |
| Sex |  |  |  |
| Male | 646 (58.09) | 980 (54.38) | 0.05 |
| Female | 466 (41.91) | 822 (45.62) |  |
| Ethnicity |  |  |  |
| Caucasian | 1,005 (90.38) | 1,657 (91.95) | 0.14 |
| Other | 107 (9.62) | 145 (8.05) |  |
| Comorbidity status |  |  |  |
| HTA | 546 (49.10) | 921 (51.11) | 0.29 |
| Diabetes | 161 (14.48) | 274 (15.21) | 0,59 |
| COPD | 607 (54.59) | 1,063 (58.99) | 0.02 |
| Coronary Artery Disease | 151 (13.58) | 349 (19.37) | <0.0001 |
| Chronic renal failure | 34 (3.06) | 70 (3.88) | 0.24 |
| Other comorbidities^†^ | 67 (6.03) | 127 (7.05) | 0.28 |
| Smoking status |  |  |  |
| Never smoker | 96 (8.64) | 103 (5.72) | 0,03 |
| Former smoker | 790 (71.04) | 1,327 (73.64) |  |
| Current smoker | 226 (20.32) | 372 (20.64) |  |
| Pack-years of smoking |  |  |  |
| Never smoker | 96 (9.13) | 103 (6.02) | 0.01 |
| 1 to 20 pack-years | 178 (16.94) | 274 (16.02) |  |
| 21 to 40 pack-years | 365 (34.73) | 557 (32.57) |  |
| >40 pack-years | 412 (39.20) | 776 (45.38) |  |
| Pulmonary function test |  |  |  |
| FVC ≥80% | 899 (80.85) | 1,410 (78.25) | 0.09 |
| FVC <80% | 213 (19.15) | 392 (21.75) |  |
| FEV_1_ ≥80% | 667 (59.98) | 1,027 (56.99) | 0.11 |
| FEV_1_ <80% | 445 (40.02) | 775 (43.01) |  |
| DLCO ≥80% | 617 (55.49) | 1,022 (56.71) | 0.52 |
| DLCO <80% | 495 (44.51) | 780 (43.29) |  |
| Pathological type |  |  |  |
| Adenocarcinoma | 760 (68.35) | 1,197 (66.43) | 0,55 |
| Squamous cell carcinoma | 205 (18.44) | 357 (19.81) |  |
| Other^‡^ | 147 (13.22) | 248 (13.76) |  |
| Cancer stage |  |  |  |
| Stage 1 | 765 (78.54) | 1,118 (75.08) | 0,12 |
| Stage 2 | 145 (14.89) | 236 (15.85) |  |
| Stage 3A | 52 (5.34) | 112 (7.52) |  |
| Stage 3B/4 | 12 (1.23) | 23 (1.54) |  |
| Surgery type |  |  |  |
| Pneumonectomy | 56 (5.04) | 122 (6.77) | <0.0001 |
| Lobectomy/bilobectomy | 832 (74.82) | 1,349 (74.85) |  |
| Sublobar resection | 144 (12.95) | 331 (18.37) |  |
| Others^§^ | 80 (7.19) | 0 (0.00) |  |

DLCO= diffusing capacity of the lung for carbon monoxide; FEV_1_ = post-bronchodilator forced expiratory volume in one second; FVC = forced vital capacity;

**^*^**P-values calculated using Pearson's chi-square test, Fisher's exact test, or the Wilcoxon rank-sum test.

^†^: Other comorbidities include pulmonary embolism, pulmonary hypertension and connectivitis.

^‡^: Other pathological types include, adenosquamous carcinoma, large cell carcinoma, basaloid carcinoma, sarcomatoid carcinoma, as well as mixed tumors (i.e., multiple histological subtypes in the same lesion). Small cell lung cancers, carcinoid tumors and benign tumors were excluded of the study.

§:Other surgery types include open pulmonary biopsy, pleural biopsy, pleurectomy, decortication and talcage

**Table S2**. Association between imaging pattern and mortality in patients who underwent surgery for lung cancer and survived the 30 days postoperative period.

|  |  | **Crude HR** | | | **Adjusted HR^*^** | | | |
| --- | --- | --- | --- | --- | --- | --- | --- | --- |
|  | deaths/person-years | HR | 95% CI | P value | aHR^*^ | | 95% CI | P value |
| Imaging pattern |  |  |  |  |  |  | |  |
| No ILA | 442/7,380.72 | Ref | - | - | - | - | | - |
| ILA | 33/393.02 | 1.37 | 0.96 – 1.96 | 0.08 | 1.18 | 0.78 – 1.77 | | 0.44 |
| Non-UIP ILA | 24/346.23 | 1.13 | 0.75 – 1.71 | 0.55 | 0.94 | 0.58 – 1.51 | | 0.79 |
| UIP ILA | 9/46.80 | 3.13 | 1.62 – 6.06 | 0.001 | 3.14 | 1.50 – 6.57 | | 0.002 |

HR = Hazard ratio
^*^Adjusted analyses adjusted for age, sex, pack-years smoking, current smoking status, comorbidities, lung function, surgery type and approach (thoracoscopy or thoracotomy), pathological type and cancer stage.

**Table S3**. Association between ILA and long-term mortality in patients who underwent surgery for lung cancer according to (A) FEV1 and (B) concomitant lung obstruction.

| **A** | **FEV_1_≥80%** | | **FEV_1_<80%** | | aHR^*^ (95% CI) for FEV_1_within strata of ILA group |
| --- | --- | --- | --- | --- | --- |
|  | deaths/person-years | aHR**^†^** (95% CI) | deaths/person-years | aHR**^†^** (95% CI) |  |
| No ILA | 208/4,416.14 | 1 | 251/3,103.34 | 1.54 (1.22 – 1.94) | 1.54 (1.22 – 1.94) |
| ILA | 22/238.33 | 1.48 (0.88 – 2.49) | 15/164.02 | 1.44 (0.78 – 2.65) | 0.97 (0.46 – 2.05) |
| Non UIP ILA | 15/191.33 | 1.37 (0.75 – 2.51) | 12/162.83 | 1.11 (0.55 – 2.21) | 0.81 (0.34 – 1.93) |
| UIP ILA | 7/47.01 | 1.87 (0.75 – 4.66) | 3/1.19 | 30.68 (8.76 – 107.43) | 16.42 (3.59 – 75.06) |

| **B** | **FEV1/FVC < 0.70** | | **FEV1/FVC ≥ 0.70** | | aHR^*^ (95% CI) for lung obstruction within strata of ILA group |
| --- | --- | --- | --- | --- | --- |
|  | deaths/person-years | aHR**^†^** (95% CI) | deaths/person-years | aHR**^†^** (95% CI) |  |
| No ILA | 141/3,105.37 | 1 | 318/4414.11 | 1.01 (0.77 – 1.33) | 1.01 (0.77 – 1.33) |
| ILA | 13/124.43 | 1.36 (0.68 – 2.74) | 24/277.93 | 1.14 (0.68 – 1.90) | 0.84 (0.37 – 1.87) |
| Non-UIP ILA | 6/93.89 | 0.71 (0.26 – 1.94) | 21/260.27 | 1.09 (0.63 – 1.88) | 1.54 (0.51 – 4.66) |
| UIP ILA | 7/30.54 | 5.24 (2.05 -13.37) | 3/17.66 | 1.60 (0.49 – 5.20) | 0.31 (0.07 – 1.32) |

aHR = adjusted hazard ratio; FEV_1_ = post-bronchodilator forced expiratory volume in one second; FVC = forced vital capacity; ILA = Interstitial lung abnormalities; NC= not calculable; UIP = usual interstitial pneumonia.

^*^Adjusted analyses are adjusted for age, sex, pack-years smoking, current smoking status, comorbidities, lung function, surgery type and approach (thoracoscopy or thoracotomy), pathological type and cancer stage.
